# A new anti-malarial drug against murine malaria

**Published:** 2011-01-10

**Authors:** Zahra Zamani, Alireza Sadeghi Tafreshi, Hossein Nahrevanian, Mohammad Arjmand, Behzad Lame-Rad, Sedigheh Sadeghi, Fatemeh Mirkhani, Ali Eslamifar, Fatemeh Pourfallah, Ayda Iravani, Farideh Wahabi

**Affiliations:** 1 Department of Biochemistry, Pasteur Institute of Iran, Tehran, Iran; 2 Department of Parasitology, Pasteur Institute of Iran, Tehran, Iran; 3 Department of Clinical Reaserch, Pasteur Institute of Iran, Tehran, Iran; 4 Department of Biochemistry, Payame Noor University of Tehran, Iran

## 

Eosin B, a common laboratory dye has been earlier reported to have good anti protozoan properties *in vitro*. We studied this drug for its effect on a murine malaria strain, *Plasmodium berghei* (*Pb*) *in vivo* using different tests. Full suppressive 4 days Peter’s Test was used in infected outbred and inbred mice, using both ip and oral routes. Secondary biological assessment was carried out using dose ranging, ED50 and ED90 values obtained. Eosin B anti malarial activity at 400 Ug/ml given in both the routes was similar to that of artemisine and mice survival rate was double that of control and 3 days more than artemisine. *Pb*GST activity was monitored and it was seen that eosin B lowered this enzyme activity. Eosin B seems to be a promising drug, exhibiting good anti malarial effects in the murine model of disease.

